# Longitudinal assessment of quality-of-life after gastrectomy for gastric cancer: a prospective study

**DOI:** 10.1007/s10120-026-01805-z

**Published:** 2026-10-01

**Authors:** Shinji Kuroda, Yoshihiko Kakiuchi, Yudai Mimata, Hitoshi Minagi, Shunya Hanzawa, Hajime Kashima, Satoru Kikuchi, Toshiyoshi Fujiwara

**Affiliations:** 1 Department of Gastroenterological Surgery, Dentistry and Pharmaceutical Sciences, Okayama University Graduate School of Medicine, 2-5-1 Shikata-cho, Kita-ku, Okayama, 700-8558 Japan; 2 Kagawa Rosai Hospital, Marugame, Japan

**Keywords:** Gastric cancer, Gastrectomy, Quality of life, Patient-reported outcome, Postgastrectomy Syndrome Assessment Scale-45 (PGSAS-45)

## Abstract

**Background:**

The longitudinal recovery process for postoperative quality of life (QOL) after gastrectomy remains incompletely understood. We prospectively evaluated temporal changes in QOL using the Postgastrectomy Syndrome Assessment Scale-45 (PGSAS-45) and investigated predictors of poor postoperative QOL.

**Methods:**

This prospective observational study enrolled 433 patients who underwent gastrectomy for primary gastric cancer between 2015 and 2020. Patients completed the PGSAS-45 questionnaire preoperatively and at 1 month, 6 months, 1 year, and > 5 years after surgery. Longitudinal changes in QOL were analyzed, and multivariable logistic regression was performed to identify predictors of poor postoperative QOL at 1 year after surgery.

**Results:**

All PGSAS-45 domains except the mental component summary (MCS) were significantly deteriorated at 1 month after surgery. Most domains recovered substantially between 1 and 6 months and changed little thereafter. MCS was the only domain that improved beyond the preoperative level. Independent predictors of poor total symptom score were major postoperative complications (Clavien–Dindo grade ≥ III; odds ratio [OR] 4.78, *p* = 0.013) and preoperative MCS < 46 (OR 2.45, *p* = 0.001). Preoperative physical component summary (PCS) < 46 (OR 2.01, *p* = 0.028) independently predicted poor quality of ingestion, while preoperative MCS < 46 (OR 2.81, *p* < 0.001) independently predicted greater dissatisfaction with daily life.

**Conclusions:**

Postoperative QOL recovered predominantly within the first 6 months after gastrectomy and remained largely stable thereafter. Preoperative PCS and MCS, together with major postoperative complications, were important determinants of postoperative QOL.

**Supplementary Information:**

The online version contains supplementary material available at https://doi.org/10.1007/s10120-026-01805-z.

## Introduction

Advances in the early detection and multidisciplinary treatment of gastric cancer (GC) have substantially improved long-term survival, making the preservation of postoperative quality of life (QOL) an increasingly important therapeutic goal in addition to oncological cure [1]. Gastrectomy inevitably causes anatomical and physiological alterations that may result in postgastrectomy syndrome, nutritional impairment, and deteriorations in physical and mental well-being [2]. Consequently, patient-reported outcomes have become essential measures for evaluating the quality of GC surgery, enabling comparisons among different surgical procedures and facilitating optimization of perioperative care and long-term patient outcomes [3, 4].

The Postgastrectomy Syndrome Assessment Scale-45 (PGSAS-45) is a validated questionnaire comprising the generic 8-item Short-Form Health Survey (SF-8) and 37 items specific to postgastrectomy syndrome, enabling comprehensive assessment of both general health-related QOL and postgastrectomy-specific symptoms [5, 6]. Since its development, the PGSAS-45 has been widely used to compare postoperative QOL among different gastrectomy procedures and reconstruction methods, providing valuable evidence for selecting function-preserving surgical strategies [7–11]. Although many studies have evaluated patients at a single postoperative time point, several prospective longitudinal studies using the European Organisation for Research and Treatment of Cancer Quality of Life Questionnaire-Core 30 (EORTC QLQ-C30) and its GC-specific module (QLQ-STO22) or the PGSAS-37 have characterized changes in QOL after gastrectomy [12–17]. Nevertheless, few studies have longitudinally evaluated QOL using the PGSAS-45 from preoperatively through long-term follow-up. Further, the long-term trajectory of comprehensive QOL recovery beyond 5 years after gastrectomy and the timing at which postoperative QOL reaches a relatively stable level remain incompletely characterized.

Another clinically important issue is the identification of patients at risk of persistent postoperative impairment of QOL. Although surgical procedures and postoperative complications reportedly influence long-term QOL [18–20], little is known about the contribution of preoperative patient-related factors, particularly baseline physical and mental health status, to subsequent postoperative outcomes. Identifying such predictors would facilitate individualized perioperative management and targeted supportive care.

The present prospective longitudinal study used the PGSAS-45 questionnaire to serially evaluate QOL before surgery and at 1 month, 6 months, 1 year, and > 5 years after surgery. The aims of this study were to characterize the temporal trajectory of postoperative QOL recovery and determine independent predictors of poor postoperative QOL.

## Methods

### Study design and patients

This prospective observational study enrolled consecutive patients who underwent gastrectomy for primary GC at Okayama University Hospital between September 2015 and December 2020. Patients who were deemed unable to complete the QOL questionnaire because of cognitive impairment or other conditions, or who were otherwise considered unsuitable for participation in the study, were excluded.

Patients completed serial QOL assessments using the PGSAS-45 questionnaire preoperatively and at 1 month, 6 months, 1 year, and > 5 years after surgery. Preoperative questionnaires were completed on the surgical ward after hospital admission in the absence of the attending surgeon. At 1, 6, and 12 months after surgery, questionnaires were completed either during scheduled outpatient visits in the absence of the attending surgeon or by mail. Questionnaires administered > 5 years after surgery were completed by mail.

Clinicopathological data, including patient characteristics (age, sex, body mass index [BMI], and American Society of Anesthesiologists physical status [ASA-PS]), surgical factors (surgical approach, surgical procedure, extent of lymph node dissection [D], reconstruction method, operative time, blood loss, postoperative complications, and postoperative hospital stay), neoadjuvant chemotherapy (NAC), adjuvant chemotherapy (Adj), pathological findings (histological type, pathological tumor depth, pathological lymph node metastasis, pathological distant metastasis, and pathological stage [pStage]), and survival outcomes were prospectively collected from the institutional clinical databases. Postoperative complications were graded according to the Clavien–Dindo classification (CD) [21], and pathological findings were classified according to the 3rd English edition of the Japanese Classification of Gastric Carcinoma [22].

The study was conducted in accordance with the Declaration of Helsinki and was approved by the institutional review board of Okayama University Hospital (approval no. 1507-051). An information sheet explaining the study was provided with the questionnaire, and informed consent was implied by completion and return of the questionnaire.

### QOL assessment

The PGSAS-45 provides a comprehensive assessment of postgastrectomy symptoms and health-related QOL. The primary outcomes analyzed in the present study included total symptom score, derived from seven symptom subscale (SS) scores (esophageal reflux; abdominal pain; meal-related distress; indigestion; diarrhea; constipation; and dumping), together with the quality of ingestion SS, dissatisfaction with daily life SS, physical component summary (PCS), mental component summary (MCS), and body weight. Higher scores indicate better conditions for PCS, MCS, and quality of ingestion SS, whereas higher scores indicate worse conditions for the remaining outcomes.

Longitudinal changes in each PGSAS-45 domain were evaluated using mean ± standard deviation values obtained from the five assessment time points (preoperatively and at 1 month, 6 months, 1 year, and > 5 years after surgery). Pairwise comparisons were performed between consecutive assessment points (preoperatively vs. 1 month, 1 month vs. 6 months, 6 months vs. 1 year, and 1 year vs. >5 years).

The completion rate at each assessment time point was defined as the proportion of patients with available PGSAS-45 data among those eligible for assessment.

### Risk factor analysis

To identify factors associated with poor postoperative QOL, three clinically relevant PGSAS-45 domains (total symptom score, quality of ingestion SS, and dissatisfaction with daily life SS) were selected as endpoints. These domains were evaluated 1 year after surgery, with cutoff values primarily based on the median value of each domain.

Potential risk factors included age, sex, BMI, ASA-PS grade, surgical approach, surgical procedure, extent of lymph node dissection, reconstruction method, operative time, blood loss, postoperative complications, postoperative hospital stay, NAC, Adj, pStage, and preoperative PCS and MCS.

Variables showing values of *p* < 0.10 in univariate analyses were entered into multivariable logistic regression analysis to identify independent predictors. Odds ratios (ORs) and 95% confidence intervals (CIs) were reported only for factors showing values of *p* < 0.05 in multivariable analysis.

### Statistical analysis

Continuous variables are presented as mean ± standard deviation or median and range, as appropriate, while categorical variables are presented as number and percentage. Longitudinal changes in QOL were analyzed using paired Student’s t-tests for consecutive assessment points. In risk factor analysis, variables associated with postoperative QOL in univariate analyses (*p* < 0.10) were included in multivariable logistic regression models. Overall survival was estimated using the Kaplan–Meier method.

All statistical analyses were performed using Stata version 19 (StataCorp LLC, College Station, TX, USA). A two-sided *p*-value < 0.05 was considered statistically significant.

## Results

### Patient characteristics

Participants comprised 433 patients who underwent gastrectomy for primary GC. Median duration of follow-up was 6.0 years (range, 0.0–10.2 years) after surgery.

**Fig. 1:**
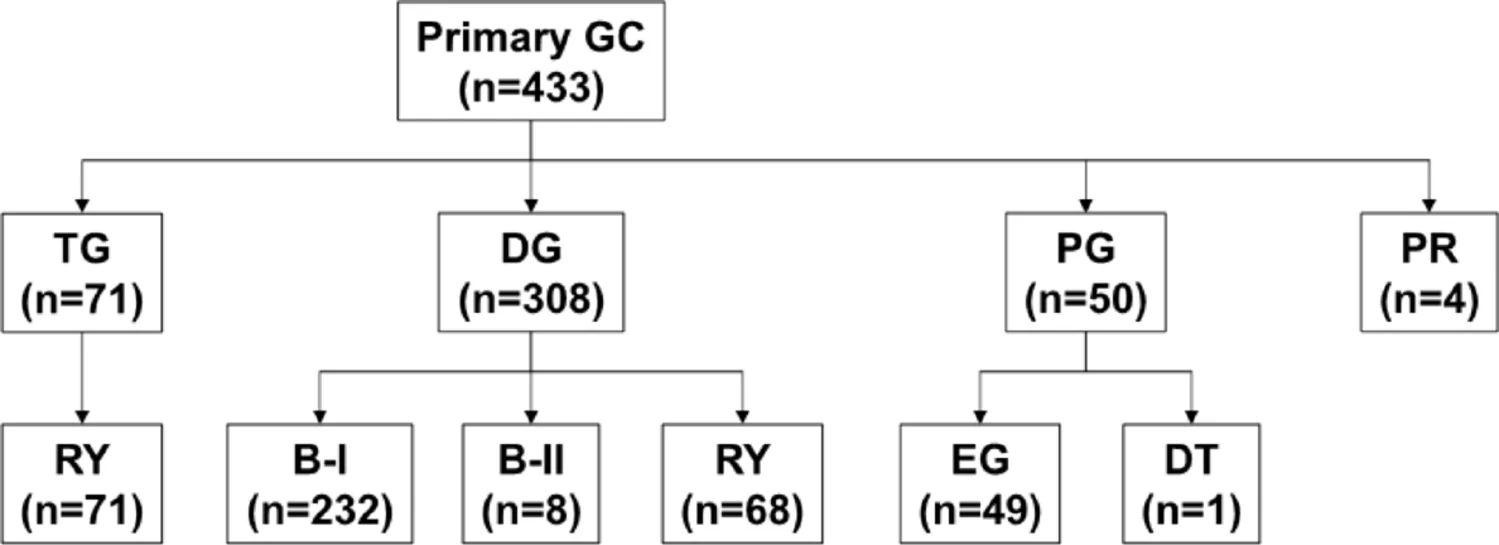
Study flow diagram. GC, gastric cancer; TG, total gastrectomy; DG, distal gastrectomy; PG, proximal gastrectomy; PR, partial resection; RY, Roux-en-Y reconstruction; B-I, Billroth-I reconstruction; B-II, Billroth-II reconstruction; EG, esophagogastrostomy; DT, double-tract reconstruction

Figure 1 illustrates the study cohort, including the distribution of surgical procedures (total gastrectomy [TG], distal gastrectomy [DG], proximal gastrectomy [PG], and partial resection [PR]) and reconstruction methods (Billroth-I [B-I], Billroth-II [B-II], Roux-en-Y [RY], esophagogastrostomy [EG], and double-tract reconstruction [DT]).

Table 1 summarizes the clinicopathological characteristics of the 433 patients included in this study. Mean age was 69.4 ± 10.6 years, and 286 patients (66%) were male. ASA-PS grade was classified as I in 118 patients, II in 239 patients, III in 73 patients, and IV in 2 patients, with data unavailable for one patient. The predominant surgical approach was laparoscopic, accounting for 365 patients (84%), while open and robotic approaches were used in 29 (7%) and 39 (9%) patients, respectively. D1 + lymphadenectomy was performed in 247 patients (57%), while D2 or more extensive lymphadenectomy was performed in 160 patients (37%). Median operative time was 303 min (range, 57–831 min) and median blood loss was 50 mL (range, 0–2265 mL). Postoperative complications (CD grade ≥ I) occurred in 102 patients (23.6%), including postoperative delirium in 15 patients, all of whom had Clavien–Dindo grade ≤II. Major postoperative complications (CD grade ≥ III) occurred in 37 patients (8.5%), including anastomotic leakage in 8 patients (1.8%), pancreatic fistula in 5 patients (1.2%), intra-abdominal abscess in 10 patients (2.3%), ileus in 6 patients (1.4%), and anastomotic stricture in 5 patients (1.2%), with some patients experiencing multiple complications. Median postoperative hospital stay was 11 days. NAC and Adj were administered to 55 (12.7%) and 106 (24.4%) patients, respectively. Pathological stage I was the most frequent stage, accounting for 291 patients (67.2%). The cohort also included 11 patients with pathological stage IV disease and 10 patients who underwent R1/R2 resection.

**Table 1:** Clinicopathological characteristics

	(n=433)
Patient characteristics	
Age, years, mean ± SD	69.4±10.6
Sex, male/female	286/147
BMI, kg/m^2^, mean ± SD	22.7±3.4
ASA-PS, 1/2/3/4/unknown	118/239/73/2/1
Surgical factors and clinical course	
Approach, open/laparoscopic/robotic	29/365/39
Surgical procedure, TG/DG/PG/PR	71/308/50/4
Extent of lymph node dissection, D0/D1/D1+/D≥2	5/21/247/160
Reconstruction, B-I/B-II/RY/EG/DT/not applicable (PR)	232/8/139/49/1/4
Operative time, min, median (range)	303 (57–831)
Blood loss, mL, median (range)	50 (0–2265)
Postoperative complications	
CD grade ≥I	102 (23.6%)
CD grade ≥III	37 (8.5%)
Anastomotic leakage	8 (1.8%)
Pancreatic fistula	5 (1.2%)
Intra-abdominal abscess	10 (2.3%)
Ileus	6 (1.4%)
Anastomotic stricture	5 (1.2%)
Bleeding	4 (0.9%)
Lymphatic/chylous leakage	4 (0.9%)
Others	4 (0.9%)
Postoperative hospital stay, days, median (range)	11 (7–94)
NAC	55 (12.7%)
Adj	106 (24.4%)
Tumor factors	
Histological type, Diff/Undiff/Other	252/170/11
pT, 0/1/2/3/4	1/272/44/63/53
pN, 0/1/2/3	305/61/31/36
pM, 0/1	422/11
pStage, I/II/III/IV/unclassifiable	291/73/57/11/1
Residual tumor, R0/R1/R2	423/7/3

SD, standard deviation; BMI, body mass index; ASA-PS, American Society of Anesthesiologists physical status; TG, total gastrectomy; DG, distal gastrectomy; PG, proximal gastrectomy; PR, partial resection; B-I, Billroth-I; B-II, Billroth-II; RY, Roux-en-Y; EG, esophagogastrostomy; DT, double-tract; CD, Clavien–Dindo classification; NAC, neoadjuvant chemotherapy; Adj, adjuvant chemotherapy; Diff, differentiated; Undiff, undifferentiated

**Fig. 2:**
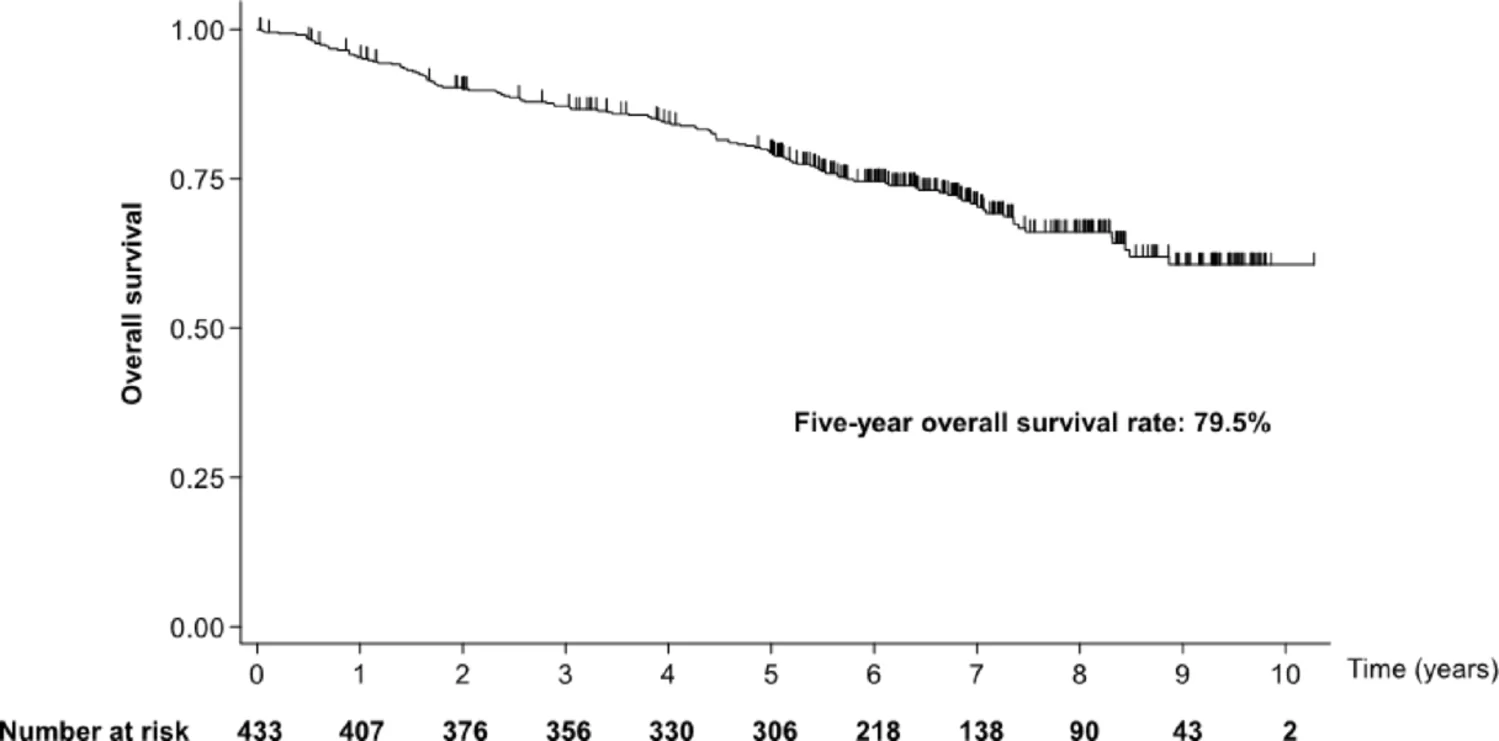
Kaplan–Meier overall survival curve

Figure 2 shows the overall survival curve constructed using Kaplan–Meier methods. The 5-year overall survival rate was 79.5% (95%CI, 75.3–83.1%), and median overall survival was not reached during follow-up.

### Longitudinal QOL changes before and after gastrectomy

The completion rate for QOL assessment was 70% (305/433) at preoperative baseline, 93% (398/430) at 1 month, 90% (382/424) at 6 months, 90% (365/407) at 1 year, and 72% (220/306) at > 5 years after surgery.

ASA-PS showed a weak negative correlation with preoperative PCS that did not reach the level of statistical significance (Spearman’s ρ = − 0.1105, *p* = 0.0568), whereas no significant correlation was observed with preoperative MCS (Spearman’s ρ = 0.0776, *p* = 0.1815).

**Fig. 3:**
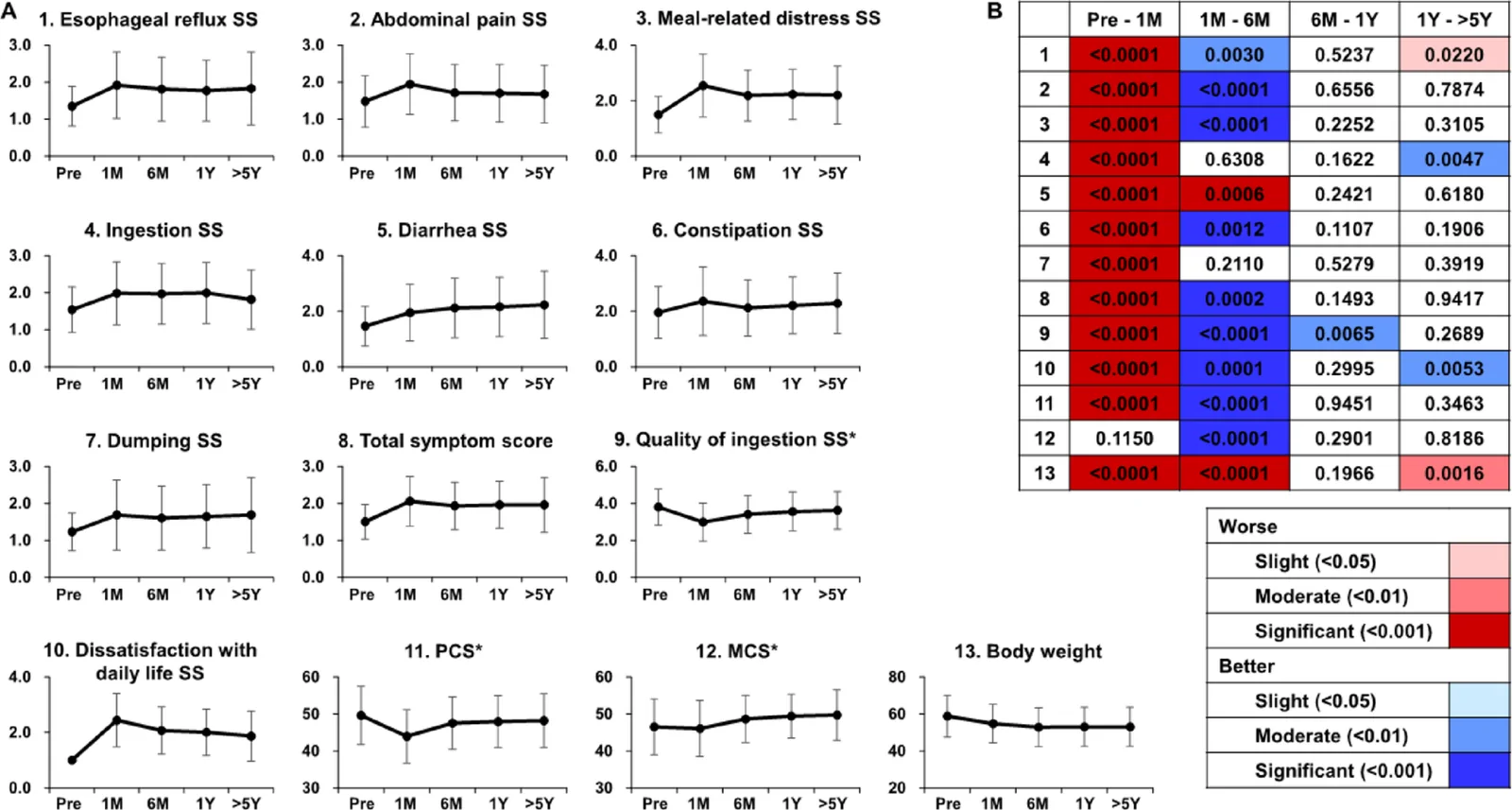
Longitudinal QOL changes assessed using the PGSAS-45. **A** Mean ± standard deviation values for each PGSAS-45 domain at five assessment time points (preoperatively and at 1 month, 6 months, 1 year, and > 5 years after surgery). Evaluated domains were: (1) esophageal reflux subscale score (SS); (2) abdominal pain SS; (3) meal-related distress SS; (4) indigestion SS; (5) diarrhea SS; (6) constipation SS; (7) dumping SS; (8) total symptom score; (9) quality of ingestion SS; (10) dissatisfaction with daily life SS; (11) physical component summary (PCS); (12) mental component summary (MCS); and (13) body weight. Asterisks indicate domains for which higher scores indicate better conditions. For all other outcomes, higher scores indicate worse conditions. **B** Pairwise comparisons between consecutive assessment time points. Numbers correspond to the domains shown in (**A**). Red indicates deterioration, blue indicates improvement, and color intensity reflects the level of statistical significance

Figure 3 illustrates longitudinal changes in postoperative QOL as assessed using the PGSAS-45 questionnaire. All QOL domains except MCS were significantly deteriorated at 1 month after surgery compared with the preoperative baseline, and significant recovery was subsequently observed between 1 and 6 months after surgery in most QOL domains except for ingestion, diarrhea, dumping, and body weight. In contrast, few significant changes were observed after 6 months. Although several domains continued to show gradual changes thereafter, the magnitude of these changes was substantially smaller than that observed during the early postoperative period.

These findings suggest that postoperative QOL reached a relatively stable level by approximately 6 months after gastrectomy in most PGSAS-45 domains, whereas only mental well-being improved beyond the preoperative level.

### Factors associated with QOL at 1 year after surgery

All seven symptom SSs correlated significantly with dissatisfaction with daily life SS, PCS, and MCS at 1 year after surgery (Supplementary Table S1). Among the individual symptoms, meal-related distress showed the strongest correlation with dissatisfaction with daily life SS (ρ = 0.5914), followed by abdominal pain (ρ = 0.5524). Abdominal pain showed the strongest correlations with PCS (ρ = − 0.4272) and MCS (ρ = − 0.4285).

Table 2 presents the results of risk factor analysis for poor total symptom score, as derived from the seven symptom SSs (esophageal reflux, abdominal pain, meal-related distress, indigestion, diarrhea, constipation, and dumping). In the univariate analyses, blood loss > 50 mL (*p* = 0.042), postoperative complications of CD grade ≥ III (*p* = 0.001), anastomosis-related complications (*p* = 0.041), postoperative hospital stay ≥ 14 days (*p* = 0.044), preoperative PCS < 46 (*p* = 0.024), and preoperative MCS < 46 (*p* = 0.001) were significantly associated with poor postoperative total symptom score, whereas TG showed only a marginal association (*p* = 0.050). In the multivariable logistic regression analysis including these seven variables, postoperative complications of CD grade ≥ III (OR 4.78, 95%CI 1.39–16.4; *p* = 0.013) and preoperative MCS < 46 (OR 2.45, 95%CI 1.43–4.22; *p* = 0.001) were identified as independent predictors of poor postoperative total symptom score.

**Table 2:** Risk factors for poor total symptom score (≥ 1.95)

	Univariate analysis	Multivariate analysis		
	*p* value	Odds ratio	95%CI	*p* value
Age (≥ 75 years)	0.885			
Sex (male)	0.947			
BMI (< 22 kg/m^2^)	0.593			
ASA-PS (≥ III)	0.509			
Approach (open)	0.993			
Surgical procedure (TG)	0.050			0.194
Extent of lymph node dissection (≥ D2)	0.326			
Reconstruction (B-I, EG)	0.330			
Operative time, min (≥ 300 min)	0.287			
Blood loss (≥ 50 mL)	0.042			0.127
CD grade (≥ III)	0.001	4.78	1.39–16.4	0.013
Anastomosis-related complications	0.041			0.923
Intra-abdominal infectious complications	0.139			
Postoperative hospital stay (≥ 14 days)	0.044			0.755
NAC	0.578			
Adj	0.829			
NAC and/or Adj	0.518			
pStage (≥ II)	0.487			
Preoperative PCS (< 46)	0.024			0.050
Preoperative MCS (< 46)	0.001	2.45	1.43–4.22	0.001

BMI, body mass index; ASA-PS, American Society of Anesthesiologists physical status; TG, total gastrectomy; B-I, Billroth-I; EG, esophagogastrostomy; CD, Clavien–Dindo classification; NAC, neoadjuvant chemotherapy; Adj, adjuvant chemotherapy; PCS, physical component summary; MCS, mental component summary; CI, confidence interval

Anastomosis-related complications include anastomotic leakage and stricture

Intra-abdominal infectious complications include anastomotic leakage, pancreatic fistula, and intra-abdominal abscess

Table 3 presents the results of risk factor analysis for poor quality of ingestion SS. In the univariate analyses, postoperative complications of CD grade ≥ III (*p* = 0.045) and preoperative PCS < 46 (*p* = 0.029) were significantly associated with poor postoperative quality of ingestion. In the multivariable logistic regression analysis including these two variables, preoperative PCS < 46 (OR 2.01, 95%CI 1.07–3.75; *p* = 0.028) was identified as an independent predictor of poor postoperative quality of ingestion.

**Table 3:** Risk factors for poor quality of ingestion SS (< 3.0)

	Univariate analysis	Multivariate analysis		
	*p* value	Odds ratio	95%CI	*p* value
Age (≥ 75 years)	0.155			
Sex (male)	0.483			
BMI (< 22 kg/m^2^)	0.924			
ASA-PS (≥ III)	0.840			
Approach (open)	0.637			
Surgical procedure (TG)	0.265			
Extent of lymph node dissection (≥ D2)	0.958			
Reconstruction (B-I, EG)	0.910			
Operative time, min (≥ 300 min)	0.328			
Blood loss (≥ 50 mL)	0.107			
CD grade (≥ III)	0.045			0.251
Anastomosis-related complications	0.107			
Intra-abdominal infectious complications	0.113			
Postoperative hospital stay (≥ 14 days)	0.749			
NAC	0.565			
Adj	0.565			
NAC and/or Adj	0.539			
pStage (≥ II)	0.394			
Preoperative PCS (< 46)	0.029	2.01	1.07–3.75	0.028
Preoperative MCS (< 46)	0.428			

BMI, body mass index; ASA-PS, American Society of Anesthesiologists physical status; TG, total gastrectomy; B-I, Billroth-I; EG, esophagogastrostomy; CD, Clavien–Dindo classification; NAC, neoadjuvant chemotherapy; Adj, adjuvant chemotherapy; PCS, physical component summary; MCS, mental component summary; CI, confidence interval

Anastomosis-related complications include anastomotic leakage and stricture

Intra-abdominal infectious complications include anastomotic leakage, pancreatic fistula, and intra-abdominal abscess

Table 4 presents the results of risk factor analysis for greater dissatisfaction with daily life SS. In the univariate analysis, TG (*p* = 0.006), pathological stage ≥ II (*p* = 0.035), and preoperative MCS < 46 (*p* < 0.001) were significantly associated with greater postoperative dissatisfaction with daily life, while postoperative complications of CD grade ≥ III (*p* = 0.057) showed a marginal association. In the multivariable logistic regression analysis including these four variables, preoperative MCS < 46 (OR 2.81, 95%CI 1.63–4.83; *p* < 0.001) was identified as an independent predictor of greater postoperative dissatisfaction with daily life.

**Table 4:** Risk factors for greater dissatisfaction with daily life SS (≥ 2.0)

	Univariate analysis	Multivariate analysis		
	*p* value	Odds ratio	95%CI	*p* value
Age (≥ 75 years)	0.599			
Sex (male)	0.876			
BMI (< 22 kg/m^2^)	0.748			
ASA-PS (≥ III)	0.713			
Approach (open)	0.625			
Surgical procedure (TG)	0.006			0.081
Extent of lymph node dissection (≥ D2)	0.227			
Reconstruction (B-I, EG)	0.367			
Operative time, min (≥ 300 min)	0.407			
Blood loss (≥ 50 mL)	0.253			
CD grade (≥ III)	0.057			0.088
Anastomosis-related complications	0.275			
Intra-abdominal infectious complications	0.678			
Postoperative hospital stay (≥ 14 days)	0.167			
NAC	0.841			
Adj	0.416			
NAC and/or Adj	0.278			
pStage (≥ II)	0.035			0.565
Preoperative PCS (< 46)	0.107			
Preoperative MCS (< 46)	< 0.001	2.81	1.63–4.83	< 0.001

BMI, body mass index; ASA-PS, American Society of Anesthesiologists physical status; TG, total gastrectomy; B-I, Billroth-I; EG, esophagogastrostomy; CD, Clavien–Dindo classification; NAC, neoadjuvant chemotherapy; Adj, adjuvant chemotherapy; PCS, physical component summary; MCS, mental component summary; CI, confidence interval

Anastomosis-related complications include anastomotic leakage and stricture

Intra-abdominal infectious complications include anastomotic leakage, pancreatic fistula, and intra-abdominal abscess

These findings suggest that both patient-related factors (including PCS and MCS) and treatment-related factors (including severe postoperative complications) play important roles in determining postoperative QOL at 1 year after gastrectomy.

## Discussion

The present prospective longitudinal study demonstrated three clinically important findings. First, postoperative QOL as assessed using the PGSAS-45 deteriorated markedly during the first postoperative month, recovered predominantly within the subsequent 6 months, and thereafter remained largely stable in most domains. Second, preoperative physical and mental health status, as represented by PCS and MCS, independently predicted poor postoperative QOL. Third, treatment-related factors, particularly severe postoperative complications, were also independently associated with impaired postoperative QOL. Collectively, these findings provide practical information regarding the optimal timing of postoperative QOL assessment and the identification of patients at increased risk for persistent postoperative QOL impairment.

One of the major strengths of the present study is the prospective longitudinal design. Although the PGSAS-45 has been widely used to compare postoperative QOL among different gastrectomy procedures and reconstruction methods, relatively few studies have serially evaluated QOL from preoperatively through long-term follow-up. In contrast, the present study serially evaluated patients from the preoperative period through more than 5 years after surgery, allowing detailed description of the postoperative recovery process. Previous prospective longitudinal studies using the EORTC QLQ-C30/QLQ-STO22 or PGSAS-37 have characterized postoperative QOL changes and compared outcomes according to surgical procedures and reconstruction methods [12–17]. Several of those studies demonstrated marked deterioration in QOL at 1 month after surgery, followed by substantial recovery by 6–12 months [12–15], a trajectory broadly consistent with that observed in the present study. One important clinical implication of our findings is that postoperative assessments of QOL performed at ≥6 months are likely to provide results comparable to those obtained at later postoperative time points in most PGSAS-45 domains. Therefore, when repeated long-term assessments are impractical, evaluation after 6 months may reasonably represent the long-term postoperative status of patients. Accordingly, the 1-year time point was selected for risk factor analysis because postoperative QOL had reached a relatively stable level while the rate of questionnaire completion still remained high. Although longer-term QOL is clinically relevant, the lower completion rate beyond 5 years could have introduced substantial attrition bias into the predictor analysis.

Another important finding of the present study is the prognostic significance of preoperative PCS and MCS. Previous studies have primarily focused on surgical procedures, reconstruction methods, and postoperative complications as determinants of postoperative QOL, whereas relatively little attention has been paid to baseline patient-related factors. Our results suggest that the baseline physical and mental health of patients substantially influence postoperative symptoms, daily functioning, and overall QOL. In particular, baseline MCS may capture individual differences in psychological responses to surgery, which could influence the perception and reporting of postoperative symptoms [23]. Further, the improvement in MCS beyond the preoperative baseline in the present study may partly reflect a “response shift,” a psychological adaptation in which patients recalibrate their internal standards, reprioritize their values, or reconceptualize QOL in response to changes in health status [24]. Such adaptation may contribute to improvements in perceived mental well-being over time despite persistent postoperative symptoms or functional changes. These findings indicate that postoperative QOL is determined not only by treatment-related factors, but also by the preoperative characteristics of patients. Preoperative assessment of PCS and MCS may therefore help identify patients at increased risk of persistent postoperative impairments in QOL as a group that may benefit from intensified nutritional, rehabilitative, or psychological support. Although the PGSAS-37 avoids the additional licensing cost associated with using the SF-8, PCS and MCS cannot be assessed. The ability to assess PCS and MCS may provide additional clinical value to the PGSAS-45. The importance of preoperative PCS and MCS also has methodological implications for future QOL research. Comparative studies evaluating different surgical procedures or reconstruction methods generally attribute postoperative differences to the treatment itself. However, because baseline PCS and MCS significantly influenced postoperative QOL in the present study, future comparative studies should evaluate these variables preoperatively and account for them through appropriate adjustment or matching to minimize patient-related confounding.

Minimally invasive gastrectomy has been actively promoted at our institution, and more than 90% of patients in the present cohort underwent laparoscopic or robotic surgery [25]. Further, the proportion of robotic gastrectomies has continued to increase in recent years [26, 27]. Regarding reconstruction, B-I is the preferred procedure after DG whenever technically feasible, whereas RY is selected when B-I proves unsuitable. After PG, EG using the double-flap technique has been adopted as the standard reconstruction because of the favorable functional outcomes [11, 28–30]. Although the present study was not intended to compare individual surgical procedures or reconstruction methods, the relatively standardized surgical strategy employed at our institution may have reduced treatment-related heterogeneity and facilitated evaluation of the natural postoperative QOL trajectory.

The present study showed several limitations. First, this was a single-center study, and our institutional treatment strategies may have limited the generalizability of the findings. *The present cohort predominantly comprised patients with relatively early-stage GC*,* reflecting the clinical setting in Japan. In populations with a higher proportion of advanced GC and substantial tumor-related symptoms*,* preoperative QOL and its postoperative trajectory may differ from those observed in the present study. Therefore*,* caution is warranted when extrapolating our findings to populations with different disease-stage distributions and symptom burdens.* Second, although the questionnaire completion rate remained high during the first postoperative year, the response rate declined beyond 5 years after surgery, potentially introducing response bias. Third, because the primary objective of this study was to characterize the overall postoperative recovery trajectory, detailed subgroup analyses were beyond the scope of the investigation. In the risk factor analysis, surgical procedures were dichotomized into TG and non-TG based on the substantial impact of total loss of the stomach on postoperative QOL. However, this classification may not fully account for procedure-specific differences among stomach-preserving procedures. Further subgroup analyses may identify distinct recovery patterns among different patient populations and provide more individualized information for clinical decision-making. Ongoing analyses are evaluating the impact of surgical procedures, reconstruction methods, age, and perioperative chemotherapy on postoperative QOL.

In conclusion, this prospective longitudinal study demonstrated that most recovery in postoperative QOL occurred within the first 6 months after gastrectomy and that QOL assessments performed at ≥6 months are likely to provide information comparable to that obtained at later postoperative time points in most PGSAS-45 domains. Furthermore, baseline physical and mental health status and severe postoperative complications were identified as important determinants of postoperative QOL. These findings may contribute to optimizing postoperative follow-up strategies, improving individualized perioperative care, and informing the design of future studies evaluating QOL after gastrectomy.

## Supplementary Information

Below is the link to the electronic supplementary material.

Supplementary Material 1
