# Supplementary material for: Longitudinal assessment of quality-of-life after gastrectomy for gastric cancer: a prospective study
Source: Gastric Cancer. 2026 Oct 1;29(6):1396–404. doi: 10.1007/s10120-026-01805-z (PMC13754761; doi:10.1007/s10120-026-01805-z)
Supplement: Supplementary file 1 [file 10120_2026_1805_MOESM1_ESM.pdf]

Supporting Information

Longitudinal Assessment of Quality-of-Life After Gastrectomy for Gastric Cancer: A Prospective Study

Shinji Kuroda[1], Yoshihiko Kakiuchi[1], Yudai Mimata[1], Hitoshi Minagi[1], Shunya Hanzawa[1], Hajime Kashima[1], Satoru Kikuchi[1], Toshiyoshi Fujiwara[1,2]

[1] Department of Gastroenterological Surgery, Okayama University Graduate School of Medicine, Dentistry and Pharmaceutical Sciences, Okayama, Japan

[2] Kagawa Rosai Hospital, Marugame, Japan

Corresponding author: Shinji Kuroda

Department of Gastroenterological Surgery

Okayama University Graduate School of Medicine, Dentistry and Pharmaceutical Sciences

2-5-1 Shikata-cho, Kita-ku, Okayama 700-8558, Japan

Tel: +81-86-235-7257; Fax: +81-86-221-8775, E-mail: shinkuro@okayama-u.ac.jp

Supplementary Table S1.

Correlations between individual postgastrectomy symptoms and QOL outcomes at 1 year after surgery

Supplementary Table S1. Correlations between individual postgastrectomy symptoms and QOL outcomes at 1 year after surgery

Symptom subscale (SS)	Dissatisfaction with daily life SS		PCS		MCS	
	ρ	*p* value	ρ	*p* value	ρ	*p* value
Esophageal reflux SS	0.4513	<0.0001	-0.3893	<0.0001	-0.3593	<0.0001
Abdominal pain SS	0.5524	<0.0001	-0.4272	<0.0001	-0.4285	<0.0001
Meal-related distress SS	0.5914	<0.0001	-0.3534	<0.0001	-0.3677	<0.0001
Ingestion SS	0.4369	<0.0001	-0.3192	<0.0001	-0.3403	<0.0001
Diarrhea SS	0.3034	<0.0001	-0.1880	0.0004	-0.3557	<0.0001
Constipation SS	0.3193	<0.0001	-0.2736	<0.0001	-0.3172	<0.0001
Dumping SS	0.4807	<0.0001	-0.1920	0.0003	-0.2744	<0.0001

PCS, physical component summary; MCS, mental component summary; ρ, Spearman's rank correlation coefficient.
